# Non-Alcoholic Fatty Liver Disease in Lean and Non-Obese Individuals: Current and Future Challenges

**DOI:** 10.3390/biomedicines9101346

**Published:** 2021-09-28

**Authors:** Mohammad Shafi Kuchay, José Ignacio Martínez-Montoro, Narendra Singh Choudhary, José Carlos Fernández-García, Bruno Ramos-Molina

**Affiliations:** 1 Division of Endocrinology and Diabetes, Medanta The Medicity Hospital, Gurugram 122001, Haryana, India; drshafikuchay@gmail.com; 2 Department of Endocrinology and Nutrition, Virgen de la Victoria University Hospital, Institute of Biomedical Research in Malaga (IBIMA), Faculty of Medicine, University of Malaga, 29010 Malaga, Spain; joseimartinezmontoro@gmail.com; 3 Department of Hepatology, Medanta The Medicity Hospital, Gurugram 122001, Haryana, India; docnarendra@gmail.com; 4 Department of Endocrinology and Nutrition, Regional University Hospital of Malaga, Institute of Biomedical Research in Malaga (IBIMA), Faculty of Medicine, University of Malaga, 29010 Malaga, Spain; 5 Obesity and Metabolism Laboratory, Biomedical Research Institute of Murcia (IMIB-Arrixaca), 30120 Murcia, Spain

**Keywords:** lean NAFLD, visceral adiposity, insulin resistance, gut microbiota, metabolic syndrome

## Abstract

Non-alcoholic fatty liver disease (NAFLD), which approximately affects a quarter of the world’s population, has become a major public health concern. Although usually associated with excess body weight, it may also affect normal-weight individuals, a condition termed as lean/non-obese NAFLD. The prevalence of lean/non-obese NAFLD is around 20% within the NAFLD population, and 5% within the general population. Recent data suggest that individuals with lean NAFLD, despite the absence of obesity, exhibit similar cardiovascular- and cancer-related mortality compared to obese NAFLD individuals and increased all-cause mortality risk. Lean and obese NAFLD individuals share several metabolic abnormalities, but present dissimilarities in genetic predisposition, body composition, gut microbiota, and susceptibility to environmental factors. Current treatment of lean NAFLD is aimed at improving overall fitness and decreasing visceral adiposity, with weight loss strategies being the cornerstone of treatment. Moreover, several drugs including PPAR agonists, SGLT2 inhibitors, or GLP-1 receptor agonists could also be useful in the management of lean NAFLD. Although there has been an increase in research regarding lean NAFLD, there are still more questions than answers. There are several potential drugs for NAFLD therapy, but clinical trials are needed to evaluate their efficacy in lean individuals.

## 1. Introduction

Non-alcoholic fatty liver disease (NAFLD) is a spectrum of liver conditions, ranging from (1) simple steatosis (non-alcoholic fatty liver; NAFL), with a low risk of progression; (2) non-alcoholic steatohepatitis (NASH), associated with inflammation and hepatocellular injury (characterized histologically by ballooning); and (3) advanced liver fibrosis, associated with an increased likelihood of progressing to cirrhosis and a higher risk of liver-related mortality [1,2]. Despite the fact that NAFLD has been increasing in prevalence over the past 2 decades, in parallel with the rising prevalence of obesity, it has also been noted that the prevalence of NAFLD is increasing in individuals with normal weight (defined by a body mass index, BMI <25 kg/m^2^ in Caucasians and a BMI <23 kg/m^2^ in Asians), a condition that has been defined as lean NAFLD [1]. Moreover, some studies have also coined the term non-obese NAFLD, which includes individuals with a BMI <30 kg/m^2^ in the Caucasian population and a BMI <25 kg/m^2^ in the Asian population [3,4]

According to epidemiological studies, approximately 10–20% of individuals with a diagnosis of NAFLD are lean [5]. Thus, in the United States, lean NAFLD is estimated to affect about 8 million–10 million individuals [6]. Importantly, lean NAFLD is not a benign condition, as it can progress to a more severe liver disease such as NASH and advanced fibrosis, which can further lead to cirrhosis [1]. Moreover, a number of studies indicate that individuals with lean NAFLD have an increased risk of developing type 2 diabetes mellitus (T2DM) and have increased all-cause mortality, as compared with subjects with obesity and NAFLD [6,7].

Taking all these data together, developing strategies to identify high-risk patients of developing lean NAFLD and designing effective therapeutic approaches for this condition should be considered as a health priority, since lean NAFLD, unfortunately, may go unnoticed for years, due to the absence of clinical manifestations, and be undetected until stages in which hepatic damage is advanced and the prognosis can be compromised. 

In this review, we summarize the available recent data on the epidemiology, pathogenesis, and therapeutic management for individuals with either lean or non-obese NAFLD and we also address the gaps in current knowledge and highlight future perspectives in the care and treatment of lean/non-obese NAFLD.

## 2. Prevalence of Lean/Non-Obese NAFLD 

The prevalence rates of lean/non-obese NAFLD vary widely, ranging from 3% to 30% in the world population. This variability may be attributed to several factors such as patient selection, diagnostic modalities, BMI cut-off values, and lifestyle and dietary customs of the evaluated populations [6].

In a study from the United States, in which the prevalence of NAFLD was estimated using data from the National Health and Nutritional Examination Survey III (NHANES III) database (1988–1991), Younossi et al. found that, among 11,613 eligible participants, 18.8% had NAFLD and 3.7% had lean NAFLD [7]. The overall prevalence of NAFLD among lean subjects was 9.7% (431/4457), whereas it was 28.8% (2061/7156) in non-lean subjects [7]. However, this is the oldest study that evaluated the prevalence of NAFLD in lean individuals. In a recent study, also from the United States, Zou et al. found that the overall prevalence of NAFLD was 32.3%; among those with NAFLD, 29.7% were non-obese, of which 13.6% had lean NAFLD [8]. In studies conducted on a Korean population, the overall prevalence of NAFLD was 20.1%, with a NAFLD prevalence ranging from 12.6% to 27.4% in non-obese subjects [9,10]. Kim et al. found that in Korean individuals the prevalence of NAFLD was 37.5%, with a lean NAFLD prevalence of 11% [11]. In China, the prevalence of NAFLD was 7.3% in 6905 non-obese participants [12]. In another study, among 1779 Chinese individuals with a BMI < 24 kg/m^2^, Feng et al. found that 7.5% of individuals had ultrasound-detected liver steatosis [13]. In Hong Kong, the prevalence rate of NAFLD based on proton-MRI spectroscopy (^1^H-MRS) was 14.8% in non-obese individuals [14]. In a study from Japan, Nishioji et al. found that the prevalence rate of non-obese NAFLD was 12.6% [15]. Additionally, in a biopsy-based study, among 157 lean liver donors from India, 53 (33.7%) had NAFLD [16]. A large meta-analysis of 84 studies showed that within the NAFLD population, 19.2% of subjects were lean and 40.8% were non-obese [17]. The same meta-analysis reported that, in the general population (comprising individuals with and without NAFLD), 12.1% of people had non-obese NAFLD and 5.1% had lean NAFLD [17]. A meta-analysis of 55,936 lean/non-obese individuals by Shi et al. reported that the pooled prevalence of NAFLD was 10.2% and 15.7% in the lean and non-obese population, respectively [18]. Zou et al. reported in a meta-analysis that included 155,846 non-obese participants an overall prevalence of NAFLD of 14.5% [19]. Finally, a meta-analysis of 205,307 individuals from 14 countries reported 4.1% as the global prevalence of lean NAFLD [20]. Table 1 shows the most relevant epidemiological studies of both lean and non-obese NAFLD, including the method for the NAFL diagnosis.

Of note, the majority of prevalence studies for lean NAFLD used ultrasound for the detection of liver steatosis, which is a technique that has poor diagnostic accuracy when liver steatosis is below 30%. Therefore, epidemiological studies may have underestimated the true prevalence of NAFLD as an important proportion of individuals with mild liver steatosis may have not been diagnosed as having NAFLD. Indeed, studies using more sensitive methods such as ^1^H-MRS found a higher prevalence rate of NAFLD (19.3%) [14].

The abovementioned studies evaluated the prevalence of NAFLD; most studies used ultrasonography for evaluation of NAFLD. However, a few studies also evaluated the prevalence of NASH, which is a progressive form of NAFLD. NASH is a less common condition in the general population, with an estimated prevalence in the United States of ~5% [7]. In the NHANES III study, when NAFLD participants with elevated liver enzymes and T2DM or insulin resistance were presumed to have NASH, the prevalence of NASH in non-obese subjects was estimated to be around 0.1% [7]. In an autopsy series of 351 participants, 18.5% of markedly obese subjects and 2.7% of lean subjects were found to have biopsy-proven NASH [21]. In a meta-analysis, among people with lean or non-obese NAFLD, 39% had NASH, 29.2% had significant liver fibrosis (stage ≥ 2), and 3.2% had cirrhosis [17].

Overall, the variability in the prevalence rates of NAFLD and NASH suggests that further studies, using consistent and accurate diagnostic modalities, are needed to provide the true prevalence of lean NAFLD/NASH worldwide. Furthermore, as liver-related and overall mortality in individuals with NAFLD highly depends on the fibrosis stage, a precise estimation of the global prevalence of advanced liver fibrosis in non-obese subjects is needed. Furthermore, longitudinal biopsy-based studies evaluating the progression of each condition of NAFLD spectrum, such as simple steatosis, NASH, and fibrosis, could help evaluate the clinical relevance and outcomes of these conditions in lean/non-obese NAFLD.

## 3. Mechanisms Underlying Lean and Non-Obese NAFLD

**Figure 1:**
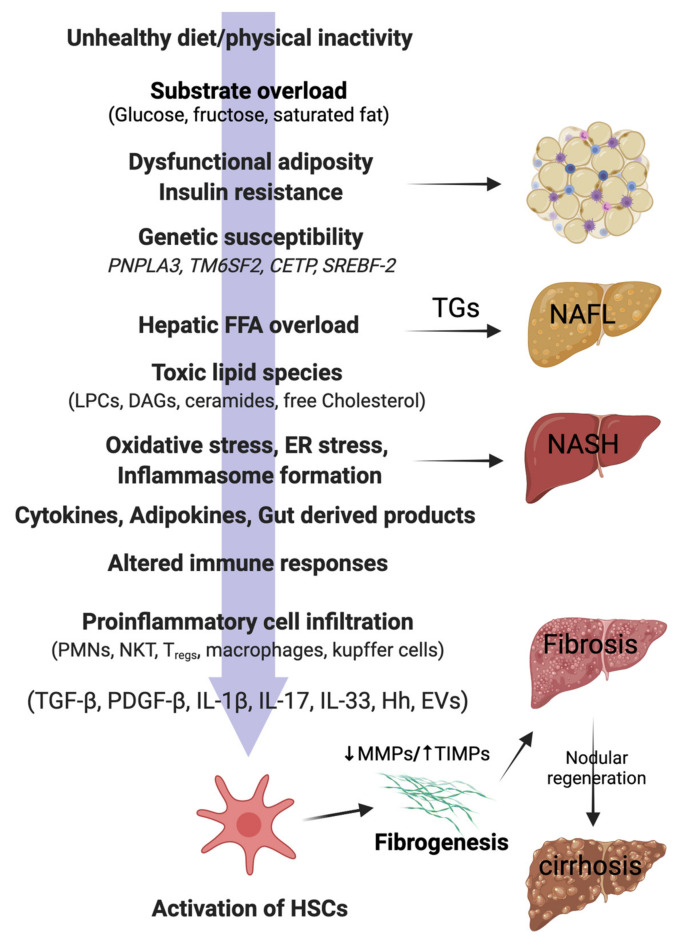
Pathophysiology of NAFLD in lean individuals. In genetically predisposed (*PNPLA3, TM6SF2*) lean or non-obese individuals, free fatty acids (FFAs), mainly derived from the visceral adipose tissue, are taken up by the liver. Hepatic FFA accumulation can be influenced by the presence of insulin resistance, dysfunctional adiposity, and/or and lifestyle habits (unhealthy diets, physical inactivity). Excess of dietary sugars (glucose, fructose) are converted into FFAs by *de novo* lipogenesis (DNL). FFAs in the liver mitochondria undergo β-oxidation or are converted back into triglycerides (TGs) for export to the circulation via VLDL. Overwhelming of FFA disposal mechanisms leads to accumulation of TGs as lipid droplets in the hepatocytes (non-alcoholic fatty liver; NAFL). When the FFA pool expands further, cytotoxic lipid species (e.g., LPCs, DAGs, and ceramides) are produced, which ultimately mediate oxidative stress, endoplasmic reticulum (ER) stress, and inflammasome activation. These pathological processes lead to hepatocellular injury, inflammatory cell recruitment, and apoptosis/necroptosis to produce the histological phenotype of non-alcoholic steatohepatitis (NASH). Major modulators of the hepatocellular response to lipotoxic stress may include the gut microbiota products; a variety of cytokines, chemokines, and adipokines; free cholesterol; uric acid; and possibly periodic hypoxia caused by obstructive sleep apnea. Overinduction of inflammatory processes then stimulates hepatic stellate cells (HSC) and activates fibrogenesis. HSC activation is the final common pathway for a diverse group of signals, such as transforming growth factor-beta (TGF-β), platelet-derived growth factor-beta (PDGF-β), interleukins (ILs), hedgehog ligands (Hhs), and extracellular vesicles (EVs). Increased tissue inhibitors of matrix metalloproteinases (TIMPs) cause inhibition of matrix metalloproteinases (MMPs), thereby leading to net gain of fibrosis tissue by the liver. Excessive and disorganized fibrous tissue causes disruption of hepatocellular architecture and nodule formation, leading to cirrhosis of the liver. *PNPLA3*, patatin-like phospholipase domain containing 3; *TM6SF2*, Transmembrane 6 superfamily 2; CETP, Cholesteryl ester transfer protein; *SREBF-2*, sterol regulatory element binding transcription factor 2; LPCs, lysophosphatidylcholines; DAGs, diacyl glycerols; PMNs, polymorphonuclear leucocytes; NKTs, natural killer T cells; T_regs_, regulatory T cells.

It is important to note that lean and non-obese NAFLD share several pathophysiological mechanisms with obese NAFLD, as illustrated in Figure 1. However, there are unique features underlying lean and non-obese NAFLD (although still not fully understood) that are described as follows.

### 3.1. Hepatic Lipid Accumulation and Lipotoxicity

The pathophysiology of lean/non-obese NAFLD is similar to obese NAFLD, with the accumulation of free fatty acids (FFAs) in the liver constituting a key process. In this line, the three major sources of hepatic fatty acids are (1) adipose tissue lipolysis, which is the source of around 60% of FFAs present in the circulation (and even higher under insulin resistance conditions) [2]; (2) excessive carbohydrates levels (especially glucose and fructose), which are converted into FFAs by the liver (*de novo* lipogenesis) and account for about 26% of stored triglycerides; and (3) dietary lipids, which constitute around 15% of triglycerides in the liver [22]. The liver deals with these FFAs’ overload by two mechanisms: (1) FFAs β-oxidation in the mitochondria and (2) re-esterification to triglycerides and subsequent export as very-low density lipoprotein (VLDL) particles. When these disposal mechanisms are overwhelmed, liver stores excess FFAs as triglycerides, leading to hepatic steatosis [2]. Furthermore, excessive β-oxidation leads to formation of reactive oxygen species (ROS) and toxic lipid species, leading to mitochondrial dysfunction, oxidative and endoplasmic reticulum (ER) stress, and inflammasome activation, finally promoting fibrogenesis [23] (Figure 1).

### 3.2. Insulin Resistance

Insulin resistance is not only a major factor underlying obese NAFLD, but also plays a key role in lean/non-obese NAFLD [13,24]. Insulin resistance contributes to NAFLD directly by increasing de novo lipogenesis (DNL) and indirectly by increasing FFAs’ delivery to the liver via decreased inhibition of lipolysis in the fat depots. In the liver, hyperinsulinemia (secondary to insulin resistance) increases the expression and activity of the transcription factor, sterol response element binding protein 1-c (*SREBP-1c*), which further increases the expression of all key enzymes required for DNL. These processes lead to accumulation of excess FFAs in the liver [22,23], which further worsens hepatic insulin resistance. This process is mediated by translocation of the PKC-δ isoform from the cytosolic to the membrane compartment, resulting in impairment of hepatic insulin receptor substrate (IRS)-associated phosphatidylinositol 3-kinase (PI3K) activity [25]. The excess of hepatic FFAs is a major driver of NAFLD pathogenesis, secondary to overwhelming of FFA disposal mechanisms and generation of toxic lipid species, such as lysophosphatidylcholines (LPCs), diacylglycerol (DAG), and ceramides. In biopsy-proven NAFLD individuals, circulatory FFAs were significantly elevated in lean, overweight, and obese NAFLD subjects, as compared with healthy controls, with myristic acid (14:0) and palmitoleic acid (16:1) being potential markers for early diagnosis of NAFLD in lean individuals [26]. So, FFAs contribute to peripheral and hepatic insulin resistance, and insulin resistance then leads to further accumulation of hepatic FFAs, thus propagating the vicious cycle.

### 3.3. Visceral Fat and Metabolic Dysfunction

Distribution of different adipose tissue depots among individuals with similar BMI is a critical factor implicated in metabolic status. Thus, several studies have reported that the contribution of visceral fat to NAFLD is more important than total body fat [13,27,28]. In this line, lean/non-obese individuals with NAFLD have relatively increased amounts of visceral adipose tissue (VAT), as compared with healthy controls [13,27,28]. A recent study noted that a higher visceral-to-subcutaneous fat ratio is associated with increased risk of NAFLD development and advanced fibrosis risk in obese/non-obese NAFLD subjects. VAT is metabolically more active than other adipose tissue depots, and it has been shown to be the source for around 5–10% of FFAs that reach the portal vein blood in lean healthy individuals. Importantly, in people with expanded visceral adiposity, the contribution of VAT to portal vein FFAs’ levels can go up to 50% [29]. Recently, a biopsy-based study including 250 lean, potential living liver donors, the severity of NAFLD was positively correlated with visceral fat accumulation [30].

VAT also constitutes a major source of adipocytokine secretion that contributes to systemic inflammation [31]. Although the specific inflammatory pathways in lean NAFLD pathophysiology are yet to be elucidated, a number of cytokines have been implicated in the development and progression of the disease. Like obesity-related NAFLD, lean individuals with NAFLD present decreased levels of adiponectin, an important hormone with insulin-sensitizing and anti-inflammatory effects [32,33]. Additionally, results from animal models reveal that interleukin 6 (IL-6) overexpression may not only be a consequence but also a central causal factor of NAFLD regardless of the presence of overweight/obesity [34]. Other potential cytokines involved in lean/non-obese NAFLD development are shown in Figure 1.

### 3.4. Sarcopenia

Low skeletal muscle mass and reduced function, termed as sarcopenia, is another clinical characteristic that may trigger NAFLD in normal-weight individuals. Moreover, several studies have reported the association of sarcopenia with NAFLD complications, such as NASH and liver fibrosis, independently of obesity [35,36,37,38,39,40]. Skeletal muscle mass seems to be lower in lean individuals with NAFLD, as compared with obese people with NAFLD [41,42]. In 762 individuals with biopsy-proven NAFLD, skeletal muscle mass was significantly lower in non-obese patients [42]. From Japan, sarcopenic obesity was significantly associated with non-obese NAFLD, and the association persisted even after adjusting for metabolic confounders (OR, 2.367; 95% CI, 1.317–4.254, *p* = 0.004) [43]. The same authors demonstrated a significant association of osteosarcopenic obesity with non-obese NAFLD in females [44]. However, the direction of cause-and-effect relationship is unknown.

Insulin resistance contributes to loss of lean body mass (sarcopenia) by the activation of ubiquitin-proteasome proteolytic pathway (UPP) in skeletal muscle. Insulin resistance reduces the activity of PI3K, which, in turn, reduces the levels of phosphorylated Akt. The decrease in phosphorylated Akt levels induces the expression and activity of E3 ubiquitin-conjugating enzymes, thereby activating UPP [45].

The muscle is the primary organ responsible for insulin-mediated glucose disposal; hence, a decrease in muscle mass may cause impairment in glucose metabolism [46]. Sarcopenia also decreases the tolerance to exercise, further decreasing energy expenditure and promoting weight gain and insulin resistance [47]. Myokines may play a role in the development in NAFLD, especially in lean and non-obese individuals. In this line, exercise stimulates the expression of peroxisome proliferator-activated receptor γ (*PPAR-γ*) coactivator-1α (*PGC-1α*), which is accompanied by greater fibronectin type III domain-containing 5 transmembrane receptor (*FNCD5*) membrane expression. FNDC5 is cleaved, releasing irisin, an exercise-inducible myokine that causes browning of white adipose tissue, increasing energy expenditure due to heat loss and weight loss [48]. In the liver, irisin has direct anti-steatogenic effects through activation of *PPAR-α* and upregulation of fibroblast growth factor 21 (*FGF21*) [49,50]. Several studies [51,52], but not all [53], have shown a negative correlation between irisin and hepatic steatosis severity.

Myostatin is another myokine that is downregulated by exercise [54]. Myostatin is an inhibitor of muscle growth since it promotes proteolysis and inhibits muscle regeneration and function [55]. Myostatin also has metabolic actions, promoting adipose tissue expansion through direct effects on the adipose tissue and indirectly through downregulation of irisin [56,57]. High myostatin concentration is associated with insulin resistance, which, in turn, is associated with muscle atrophy, decreased exercise capacity, and metabolic defects [58]. Myostatin has been shown to negatively correlate with lean mass in healthy adults [59]. Lastly, myostatin has fibrogenic properties through direct action on hepatic stellate cells [60]. Interestingly, myostatin levels correlate with liver steatosis in lean subjects [41].

On the other hand, chronic inflammation due to abnormal cytokine production in NAFLD and ROS overload have also deleterious effects on skeletal muscle, especially in lean/non-obese NAFLD. Chronic exposure to IL-6 leads to increased muscle catabolism and atrophy [61]. However, further studies are needed to establish this observation. Elevated levels of tumor necrosis factor alpha (TNF-α) induce ceramide accumulation that has been observed to contribute to muscle–cell atrophy [62]. C-reactive protein levels (CRP) correlate with the loss of total appendicular skeletal muscle [63]. Additionally, there is a well-known interplay between insulin resistance and sarcopenia. In this context, the complex crosstalk between the liver and the muscle results in a vicious circle with equally harmful consequences for both organs.

Finally, it is important to highlight that visceral adiposity and sarcopenia seem to act synergistically in the pathogenesis and progression of NAFLD. Thus, in patients with NAFLD, a decrease in muscle mass and increase in VAT are associated with worsening of steatosis and progression of liver fibrosis [64]. In this line, the assessment of body composition through different methods (e.g., bioelectrical impedance analysis (BIA), ultrasound, computed tomography (CT), magnetic resonance imaging (MRI)) could become a useful tool to detect high-risk patients. Thus, in a longitudinal cohort including almost 10,000 subjects without NAFLD (mean BMI of 22.3 kg/m^2^), body composition measurement by BIA showed that, at baseline, the lowest and middle weight-adjusted skeletal muscle index (SMI) tertiles and an increased fat percentage were associated with incident NAFLD in individuals with normal weight, while the SMI increase between examinations was an independent protective factor against NAFLD [65].

### 3.5. Genetic Predisposition

Variation in the patatin-like phospholipase domain-containing 3 (*PNPLA3*) gene is strongly linked to differences in liver fat content and susceptibility to NAFLD in lean and non-obese individuals [14,32,66,67]. *PNPLA3* is expressed in both adipocytes and hepatocytes and has acyl hydrolase activity, which leads to the hydrolysis of monoacylglycerol, diacylglycerol, and triacylglycerol [68]. The rs738409 polymorphism is associated with the loss of protein’s hydrolyzing function, thereby resulting in liver fat accumulation and insulin resistance, irrespective of body weight [69]. Indeed, a meta-analysis reported that the rs738409 polymorphism is more prevalent in non-obese/lean NAFLD patients than in obese NAFLD and non-obese controls [19].

On the other hand, genome-wide association studies found a link between NAFLD and *TM6SF2* [70,71]. Thus, the *TM6SF2* rs58542926 C > T (E167K) allele influences hepatic fibrosis irrespective of confounding risk factors such as obesity, T2DM, and age [72]. Notably, this variant has been associated with lower BMI and peripheral fat in individuals with NAFLD in phenome-wide association studies [73] Moreover, recent studies on biopsy-proven lean/non-obese NAFLD individuals reported a direct relationship between the *TM6SF2* T allele and lean/non-obese NAFLD [74,75].

Cholesteryl ester transfer protein *(CETP)* gene polymorphisms are associated with an increased risk of lean NAFLD [76]. Two single-nucleotide polymorphisms in *CETP* (rs12447924 and rs12597002) have been associated with non-obese NAFLD. The probability of lean NAFLD was above 30% in lean homozygotes, 10–15% in lean heterozygotes, and 3–5% in lean wild types, while the probability of NAFLD in obese patients was over 30% in all genotypes [76]. Furthermore, a polymorphism in the sterol regulatory element-binding factor-2 (*SREBF-2*) gene was found to be associated with lean and non-obese NAFLD [77]. In addition, it was noted that *SREBF-2* polymorphism has a significant impact on lipid and glucose metabolism and liver histology in biopsy-proven NAFLD patients and predisposes healthy individuals to develop non-obese NAFLD [77]. It was found that insufficiency of phosphatidylethanolamine N-methyltransferase (*PEMT*) increased the risk of NASH in lean individuals [78]. Another genetic factor that could play a role in lean or non-obese NAFLD is the rs368234815 TT polymorphism in the interferon lambda 4 (*IFNL4*) gene [79]. Recently, the gene of peroxisome proliferator-activated receptor-γ coactivator-1α (PGC-1α), encoded by *PPARGC1A*, was found to be a susceptibility candidate gene for NAFLD. Moreover, *PPARGC1A* rs8192678 A allele was also found to be a risk factor for non-obese NASH (OR, 22.00; 95% CI, 1.54–314.29; *p* = 0.021) [80]. However, further large studies are required to confirm the role of *CETP*, *SREBF-2*, *PEMT*, *IFNL4*, and *PPARGC1A* gene polymorphisms in the susceptibility and pathogenesis of NAFLD in lean or non-obese individuals.

It is important to highlight that gene–environment interactions (e.g., diet, physical activity, metabolic comorbidities, or gut microbiota) seem to be crucial to modulate gene polymorphism-mediated liver damage in lean/non-obese NAFLD. The low prevalence of NAFLD in lean subjects without altered metabolic profile carrying some of these mutations makes it unlikely that the presence of a risk variant itself determines the development and progression of NAFLD [81]. Thus, on a predisposed genetic background, environmental factors could trigger the disease and related complications. Intriguingly, a recent cohort study of 1339 biopsy-proven NAFLD Caucasian patients (195 lean, BMI < 25 kg/m^2^) showed that NAFLD development and progression in lean individuals were independent of their *PNPLA3* genotype [82].

In light of the many studies discussed above, further research is needed to assess the role of genetic determinants in NAFLD pathophysiology and the impact of their interplay with other risk factors.

### 3.6. Gut Microbiota Dysbiosis

Gut microbial dysbiosis is characterized by an increase in pathogenic bacteria and a decrease in number (abundance) and diversity (richness) of beneficial bacteria [83]. The mechanisms by which intestinal dysbiosis contributes to NAFLD include dysbiosis-induced gut permeability, endotoxemia, endogenous ethanol production, increased energy harvest from food, and alterations in choline and bile acid metabolism [83].

In this research line, there is growing evidence that individuals with lean NAFLD have a distinct gut microbiota profile with respect to obese individuals with NAFLD (Table 2). Thus, in a study conducted in a Chinese population, non-obese patients with NAFLD demonstrated reductions in *Firmicutes* including *Lachnospiraceae, Ruminococcaceae,* and *Lactobacillacea*, and an increase in lipopolysaccharide-producing Gram-negative bacteria [84]. Additionally, in a pilot study with biopsy-proven patients, lean NASH individuals had a lower abundance of *Faecalibacterium*, *Ruminococcus,* and *Lactobacillus* compared with non-lean individuals with NASH [85]. In another study, a decrease in *Desulfovibrionaceae* was found to be associated with lean NAFLD, compared to obese NAFLD individuals [86]. Additionally, in another study with biopsy-proven NAFLD, along with an enrichment of *Veillonellaceae* and a depletion of *Ruminococcaceae* with the worsening of liver fibrosis in individuals with non-obese NAFLD, there were increased levels of total bile acids and propionate [87]. Finally, a study conducted on a Japanese cohort of patients showed a significant decrease in the abundance of *Eubacterium* in non-obese subjects with NAFLD [88]. In another biopsy-proven cohort of 538 Caucasians patients with NAFLD, lean NAFLD subjects had higher total bile acid and FGF19 levels compared to those with non-lean NAFLD and also showed decreased levels of butyric acid [74]. Further studies are required to clearly delineate whether the changes observed in gut microbiota composition of lean individuals are either the cause or consequence of NAFLD.

## 4. Lean/Non-Obese NAFLD and Clinical Outcomes

**Figure 2:**
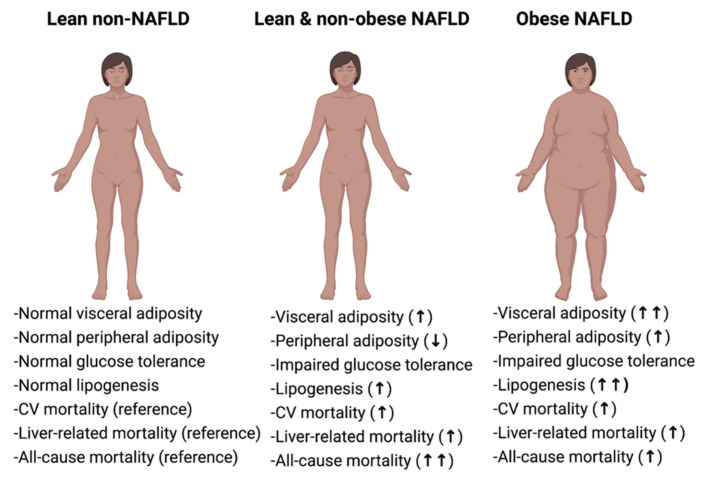
Individuals with lean and non-obese NAFLD have an intermediate phenotype between healthy (lean non-NAFLD) and obese NAFLD subjects. Lean is defined as body mass index (BMI) < 25 kg/m^2^ in Caucasians and <23 kg/m^2^ in Asians. Non-obese is defined as BMI < 30 kg/m^2^ in Caucasians and <25 kg/m^2^ in Asians. Obese NAFLD is defined as BMI ≥ 25 kg/m^2^ in Asians and ≥30 kg/m^2^ in Caucasians. NAFLD, non-alcoholic fatty liver disease; CV, cardiovascular.

Despite the fact that obese subjects with NAFLD exhibit the most deleterious pattern of metabolic derangements, individuals with lean/non-obese NAFLD have also an increased prevalence of metabolic impairment, compared to healthy controls [18,20,83,89,90,91]. In fact, lean and non-obese individuals with NAFLD seem to have an intermediate metabolic phenotype between healthy individuals and NAFLD patients with obesity [18,32,89,92] (Figure 2).

Several studies even suggest that lean individuals with NAFLD have an increased risk for incident T2DM, dyslipidemia, hypertension, cardiovascular and all-cause mortality, compared to individuals with obese NAFLD [26,91,93,94,95,96]. Individuals with lean NAFLD have a 3-fold increased risk for incident T2DM [18], even in the absence of the metabolic syndrome [93,94]. In addition, lean NAFLD patients without T2DM are also prone to develop other features related to an altered glycemic homeostasis, such as insulin resistance, and an elevated homeostatic model assessment of insulin resistance index (HOMA-IR) is often found among them [8].

### 4.1. Dyslipidaemia

Several studies found that lean and overweight/obese-NAFLD share a common lipid profile, with higher levels of triglycerides, total cholesterol, and low-density lipoprotein (LDL) cholesterol compared with both lean and overweight/obese controls [13]. In a prospective, 5-year, follow-up study including 5562 non-NAFLD subjects with a BMI < 25 kg/m^2^, triglyceride and high-density lipoprotein (HDL) cholesterol levels were associated with the presence and the development of NAFLD [12]. Moreover, in this study, LDL cholesterol levels were even higher in lean compared with overweight/obese NAFLD patients, with this finding potentially linked to a disturbance of cholesterol metabolism and a higher dietary cholesterol consumption in this population [12].

### 4.2. Hypertension

Hypertension is also a common comorbidity in both lean and overweight/obese-NAFLD. Prevalence of hypertension in lean NAFLD is significantly higher as compared to lean healthy subjects [7] and this prevalence increases with the presence of NASH [97]. In a prospective community cohort study, although lean NAFLD patients had a lower prevalence of hypertension compared to non-lean NAFLD, lean and non-lean NAFLD presented a similar risk for development of hypertension and other metabolic comorbidities [66].

Overall, a meta-analysis of 45 studies reported a lower prevalence of hypertension, and decreased uric acid and fasting glucose concentrations, but higher levels of HDL cholesterol, in lean and non-obese NAFLD population, as compared with non-lean/obese patients with NAFLD [18]. In another meta-analysis of 15 studies, lean individuals with NAFLD exhibited the entire spectrum of the metabolic syndrome: increased fasting plasma glucose, elevated insulin resistance, elevated blood lipids, and increased blood pressure and waist circumference, when compared with lean controls [92].

### 4.3. Other Clinical Outcomes

Regarding hard outcomes, an analysis of the NHANES III survey showed that, compared to healthy lean subjects, lean patients with NAFLD had a 50% increase in all-cause mortality and over a 2-fold increase in cardiovascular mortality [95]. In this line, individuals with lean NAFLD had a significantly higher atherosclerotic cardiovascular disease (ASCVD) score (defined as an ASCVD risk of >10%), 51.6% vs. 39.8% in obese NAFLD and 25.5% in subjects without NAFLD [30]. Additionally, in a recent meta-analysis from the US, non-obese NAFLD individuals had higher 15-year cumulative all-cause mortality (51.7%) than obese NAFLD (27.2%) and non-NAFLD (20.7%) [8].

With regard to NASH/fibrosis outcomes, in a community-based study, non-obese NAFLD individuals had lower liver stiffness compared with obese NAFLD individuals (4.6 kPa vs. 5.6 kPa) despite similar ^1^H-MRS-measured liver fat content. However, the percentage of advanced liver fibrosis in this patient cohort (liver stiffness measurement >9.6 kPa) was similar [14]. In a biopsy-based study, the prevalence of NASH and the severity of inflammation and fibrosis did not differ significantly between patients with normal or increased BMI [98]. Another biopsy-based study reported that 55% of patients with normal waist circumference (a surrogate for visceral adiposity) had NASH, despite milder metabolic alterations [99]. In a Chinese cohort, similar proportions of non-obese and obese patients had NASH (43.5% vs. 51.9%) and advanced fibrosis (26.1% vs. 27.7%) [100]. In a large Japanese cohort of biopsy-proven NAFLD patients, lobular inflammation, hepatocyte ballooning, and NAFLD activity score were more prevalent in the non-obese cohort [62]. A biopsy-based study from Austria reported higher rates of NASH in lean patients (18.9%) compared with overweight (8.3%) but similar to obese (17.3%) patients [101]. Fibrosis was seen in 25.7%, 13.2%, and 24.7% of lean, overweight, and obese individuals, respectively. The rate of cirrhosis was also higher in lean patients (8.1%) compared to overweight (1.7%) and obese (2.0%) patients [101]. However, a greater cirrhosis rate among the lean patients might have indicated weight loss related to cirrhosis and not that having lean NAFLD was actually a risk factor for cirrhosis. This needs to be cleared in future studies.

## 5. Clinical Presentation and Diagnosis

Most individuals with NAFLD do not notice any symptoms in the early stages of the disease. In clinical practice, most individuals are incidentally detected as having NAFLD when tested for something else, such as a health check-up. The liver enzymes are usually normal or only slightly elevated. Some patients may present with non-specific fatigue or tiredness, general lethargy, and vague discomfort in the right-upper abdomen [102].

No specific guidelines exist for the diagnosis of NAFLD in lean individuals. In order to select the most appropriate management, a thorough diagnostic workup is necessary. In clinical practice, routine imaging such as abdominal ultrasonography is sufficient for the detection of liver fat (steatosis). However, detection of liver fat is not sufficient for overall management of the patient, as it does not give information about liver fibrosis, which is the main determinant of liver-specific and cardiovascular morbidity and mortality. Several noninvasive serological scoring systems have been developed that are useful for assessing NAFLD-related fibrosis. Simple fibrosis scores such as the Fibrosis-4 (FIB-4) and the NAFLD fibrosis score (NFS) can be useful for assessing the severity of liver fibrosis in lean patients with NAFLD [103]. However, it must be borne in mind that there are no data validating for these serological scoring systems in lean or non-obese individuals with NAFLD. In patients with inconclusive findings, transient elastography or magnetic resonance elastography can be used to assess the severity of hepatic fibrosis. Liver biopsy is reserved for patients with inconclusive results or diagnostic dilemma.

## 6. Treatment Strategies for the Management of Lean/Non-Obese NAFLD

### 6.1. Lifestyle Interventions

The mainstay of treatment for obese NAFLD is lifestyle modification in order to reduce excess body weight. Thus, it has been demonstrated that weight loss (through calorie restriction and increased physical activity) is effective in ameliorating features of NAFLD [104]. Similarly, weight loss through lifestyle modification has been demonstrated to be an effective strategy for the treatment of lean or non-obese NAFLD. In a long-term, follow-up study of a clinical trial, remission of NAFLD was achieved in 67% of non-obese individuals following lifestyle intervention [101]. In this study, the authors found that most of the participants achieved NAFLD remission with a modest weight loss of 3–10% and that non-obese people were more likely than obese individuals to maintain weight reduction and normal liver enzymes [105].

Indeed, the American Association for the Study of Liver Diseases (AASLD) guidelines state that a 3–5% weight loss improves steatosis, but a greater weight loss (7–10%) is required to improve the majority of the histopathological features of NASH, including fibrosis [106]. In an interventional study conducted in Asian patients (mean BMI 25 kg/m^2^), 97% of individuals who lost more than 10% of their body weight achieved resolution of NAFLD [103]. The same study showed that approximately 40% of patients who lost between 3–5% of their body weight achieved resolution of NAFLD [107]. Another biopsy-based study demonstrated that improvement in liver histology from weight reduction by lifestyle modification was similar between individuals with non-obese NAFLD and those with obese NAFLD [108].

Regarding the dietary approach, clinical practice guidelines recommend the Mediterranean diet as the diet of choice for all NAFLD patients [109]. The principal aspects of the Mediterranean diet are increased omega-3 and monounsaturated FA intake and decreased carbohydrate intake, refined carbohydrates, and sugars [110,111]. Adherence to this diet leads to a substantial decrease in liver steatosis even without weight reduction, which makes it an interesting choice for lean NAFLD subjects [110,111]. However, the evidence is limited to small and short-term trials, and further long-term studies are required.

Increased physical activity has also beneficial effects on NAFLD independent of weight loss. Exercise, particularly aerobic exercise, seems to preferentially target visceral adiposity over subcutaneous adiposity, and may have beneficial effects on non-obese NAFLD [112]. A large study of Asian individuals (mean BMI 23.7 kg/m^2^) showed an inverse relationship between various types of physical activity and the prevalence of NAFLD in a dose-dependent manner, which was independent of visceral adiposity and insulin resistance [113].

### 6.2. Pharmacologic Treatments

A group of therapeutic agents influencing the nuclear receptors, peroxisomal proliferator-activated receptors (PPARs), plays a pivotal role in the regulation of glucose and lipid metabolism. These receptors, PPAR-α (mainly in liver), PPAR-β/δ (mainly in skeletal muscle), and PPAR-γ (mainly in adipose tissue), are also involved in inflammatory and fibrogenic pathways in liver and other organs that all contribute to NASH pathogenesis. Therefore, these drugs could be useful in lean and non-obese NAFLD, as they address dysfunctional visceral adiposity, which significantly contributes to NASH pathogenesis in lean individuals.

Thiazolidinediones, such as pioglitazone, are PPAR-γ agonists approved for the treatment of T2DM. These agents promote adipocyte differentiation and fat cell hyperplasia in adipocytes in subcutaneous compartments and decrease visceral adiposity. Pioglitazone also improves insulin sensitivity [114]. Since visceral adiposity and insulin resistance are the central processes underlying lean and non-obese NAFLD, pioglitazone could be potentially a useful agent for lean and non-obese NAFLD individuals, in line with the benefits proven in obese-NAFLD [115].

Saroglitazar, a dual PPAR-α/γ agonist, is a potential therapeutic option for lean and non-obese NAFLD, as it addresses the two main issues of NAFLD: dyslipidemia and insulin resistance [116,117]. Indeed, it was shown that saroglitazar reduced liver fat and improved dyslipidemia in patients with T2DM [116,117]. In addition, saroglitazar improved insulin resistance and NASH in an animal model of NAFLD [118]. A pan-PARR agonist, lanifibranor, improved all histologic features of NASH in experimental models [119,120].

The therapeutic agents for the potential treatments of lean or non-obese NAFLD are summarized in Table 3. Among them, novel glucose-lowering agents may constitute an effective option in this population. Glucagon-like peptide-1 receptor agonists (GLP-1 RAs) have demonstrated important benefits in obese NAFLD patients with and without T2DM [121,122,123]. Moreover, the metabolic efficacy of these therapeutic agents seems to be similar in patients without overweight/obesity [124]. GLP-1 RAs have direct hepatic actions: They can prevent accumulation of ceramides/sphingomyelins species, inflammation, and fibrosis and also induce changes in gut microbiota [125]. In animal models of non-obese NASH, GLP-1 RAs attenuate hepatic steatosis and inflammation [126,127]. In addition, the dual glucose-dependent insulinotropic peptide and GLP-1RA tirzepatide may be effective for both lean and obese subjects with NASH, via weight loss, reduction of inflammatory parameters, and increasing adiponectin concentrations, although specific trials are needed to assess these preliminary results [128].

Sodium-glucose cotransporter 2 inhibitors (SGLT2i) decrease glucose reabsorption in the proximal tubule and may be useful for NAFLD treatment through the inhibition of DNL, inflammation, ROS production, and hepatocyte death [129]. Additionally, they could even attenuate the development of hepatocellular carcinoma, as demonstrated in animal models [130]. Several trials showed a reduction of hepatic fat content after SGLT2i treatment in patients with type 2 diabetes [131,132,133]. Interestingly, in one of these trials it was shown a reduction in liver fat and an improvement in liver enzymes independent of body weight loss [131]. Therefore, although SGLT2i has been reported to reduce VAT and improve NAFLD through several mechanisms, hepatic outcomes need to be specifically assessed in patients with lean/non-obese NAFLD.

Vitamin E is associated with NASH improvement in patients without T2DM [134]. Vitamin E is a lipophilic antioxidant that reverses liver injury and inflammation and prevents progression of NAFLD [133]. Interestingly, in an open-label, single-arm study in humans (22% of them with a BMI < 25 kg/m^2^), Vitamin E was effective in NASH improvement (assessed by non-invasive markers) despite the fact that no changes in BMI were achieved [135,136].

Farnesoid X receptor (FXR) agonists may be an interesting target for lean/non-obese NAFLD as they improve inflammation/fibrosis and target gut microbiota [137]. The FXR agonist obeticholic acid was shown to significantly improve fibrosis and NASH activity in patients with advanced NAFLD and at least one concomitant comorbidity (obesity, T2DM, or alanine amino transferase-ALT >1.5 upper limit of normal) [138].

Resmetirom, a selective thyroid hormone receptor-β agonist, may improve NASH by increasing hepatic fat metabolism and reducing lipotoxicity [139]. Finally, a pilot, randomized trial conducted in lean patients with NAFLD revealed that symbiotic supplementation and lifestyle interventions led to a greater decrease in hepatic steatosis and fibrosis (assessed by non-invasive methods) as compared to lifestyle interventions alone [140]. Thus, the restoration of the healthy microbiota composition may affect the prognosis of the disease in lean/non-obese subjects.

Finally, treatment response could be genetically driven. Indeed, the *PNPLA3* gene polymorphism could determine response to lifestyle modification, as it has been shown that individuals with *PNPLA3* rs738409 GG genotype are more sensitive to the beneficial effects of lifestyle modification than in those with homozygous CC genotype [141,142]. On the other hand, a study identified several genetic determinants independently associated with histologic response to pioglitazone in NASH, including adenosine A1 receptor (*ADORA1*) rs903361 (NASH resolution) and *PPAR-γ* rs178172176 G genotype (worse improvement in fibrosis score among Hispanic population), and a genetic response score was generated based on the main results [143]. Thus, this approach may help to select patients who could benefit from thiazolidinediones and/or other therapies.

## 7. Conclusions and Future Perspectives

Lean NAFLD is an increasing condition that worsens metabolic profile and increases all-cause mortality. As patients with lean NAFLD can suffer from the whole spectrum of liver disease, accurate work-up studies are required for evaluating the true prevalence of NASH, advanced liver fibrosis, or compensated cirrhosis. Individuals with lean/non-obese NAFLD, despite not presenting with obesity, have increased visceral adiposity, and sarcopenia is a common feature. Since both characteristics act synergistically, worsening the prognosis, the assessment of body composition could help to identify high-risk subjects. Therapeutic management of patients with lean NAFLD is based on lifestyle modifications to address increased visceral fat and insulin resistance. Future potential treatments for this condition include agents that act through PPAR mechanism, such as pioglitazone or saroglitazar. Additionally, other potential drugs for this condition could be GLP-1 RAs, SGLT2is, or FXR agonists, such as obeticholic acid, but devoted clinical trials are needed to evaluate their efficacy in lean NAFLD.

## Figures and Tables

**Table 1:** Selected prevalence studies of lean and non-obese NAFLD in adult populations.

Study, Year	Country	Population	Sample Size	Non-Obese NAFLD * Prevalence (% of Population)	Lean NAFLD ** Prevalence (% of Population)	Mode of Diagnosis	Overall NAFLD Prevalence (in Population)
Kwon et al., 2012 [10]	Korea	Hospital-based	29,994	12.6%	-	USG	20.1%
Younossi et al., 2012 [7]	USA	NHANES III database (1988–1991)	11,613	-	3.7%	USG	18.8%
Sinn et al., 2012 [9]	Korea	Non-obese population	5878	27.4%	16%	USG	-
Xu et al., 2013 [12]	China	Non-obese population	6905	7.27%	-	USG	-
Feng et al., 2014 [13]	China	Annual health check-ups	1779	-	7.5%	USG	50.5%
Nishioji et al., 2015 [15]	Japan	Health check-ups	3271	12.6%	-	USG	24.6%
Wei et al., 2015 [14]	Hong Kong	Urban general population	911	14.8%	-	^1^H-MRS	28.8%
Ye et al., 2020 [17]	Global	Global	10,530,308	12.1%	5.1%	Mainly USG	-
Zou et al., 2020 [19]	USA	General population	14,365	9.6%	1.3%	USG/fatty liver index	32.3%
Lu et al., 2020 [20]	Global	Global	205,307	-	4.1%	Mainly USG	-
Kim et al., 2021 [11]	Korea	General, KNHANES (2008–2010)	4786	-	11%	Comprehensive NAFLD score	37.5%
Shi et al., 2020 [18]	China	Lean/non-obese	55,936	15.7%	10.2%	Mainly USG	-

* Non-obese is defined as <25 kg/m^2^ in Asians and <30 kg/m^2^ in Caucasians. ** Lean is defined as BMI < 23 kg/m^2^ in Asian populations and <25 kg/m^2^ in Caucasians. KNHANES: Korean National Health and Nutrition Examination Survey. ^1^H-MRS: proton magnetic resonance spectroscopy; NHANES III: National Health and Nutrition Examination Survey III; NAFLD: non-alcoholic fatty liver disease; USG: ultrasonography.

**Table 2:** Clinical studies of gut microbiota in individuals with lean NAFLD.

Study, Year	Subjects	Diagnosis of NAFLD	Lean or Non-Obese	Decreased Abundance Associated with NAFLD	Increased Abundance Associated with NAFLD
Wang et al., 2016 [84]	126 non-obese subjects	USG	Non-obese	*Lachnospiraceae* *Ruminococcaceae* *Lactobacillaceae*	LPS-producing Gram negative bacteria
Duarte et al., 2018 [85]	13 NASH; 10 controls	Biopsy	Lean	*Faecalibacterium* *Ruminococcus* *lactobacillus*	-
Yun et al., 2019 [86]	268 health check-up examinees	USG	Non-obese	*Desulfovibrionaceae*	-
Lee et al., 2020 [87]	171 Asians	Biopsy	Non-obese	*Ruminococcaceae*	*Veillonellaceae*
Iwaki et al., 2021 [88]	51 non-obese NAFLD; 51 obese NAFLD; 87 controls	Biopsy	Non-obese	*Eubacterium*	-

LPS, lipopolysaccharide; NAFLD, non-alcoholic fatty liver disease; USG, ultrasonography.

**Table 3:** Potential therapeutic agents for the treatment of lean or non-obese NAFLD.

Drug	Type	Mechanism	Side Effects
Pioglitazone	PPAR-γ agonist	Increase free fatty acid oxidation Improve insulin resistance Anti-inflammatory Anti-fibrotic	Weight gain Fluid retention (edema) Osteoporosis (bone fracture) Minimal risk of bladder carcinoma
Saroglitazar	PPAR-α/γ agonist	Increase free fatty acid oxidation Improve insulin resistance Anti-inflammatory Anti-fibrotic	Headache Gastritis (Nausea, vomiting) Fever
Lanifibranor	Pan-PPAR agonist	Increase free fatty acid oxidation Improve insulin resistance Anti-inflammatory Anti-fibrotic	Diarrhea Nausea Fatigue
Empagliflozin Dapagliflozin Canagliflozin	SGLT-2 inhibitors	Reduce glucose delivery to liver by diverting through urine	Genitourinary infections Dyslipidemia
Liraglutide Dulaglutide Semaglutide	GLP-1 receptor agonists	Improve insulin resistance Reduce food intake Induce weight loss	Upper gastrointestinal upset (nausea, vomiting) Diarrhea Weakness Pancreatitis
Vitamin E	-	Anti-oxidant	Nausea Headache Blurred vision
Resmetirom	Thyroid hormone receptor β mimetic	Increase hepatic fat metabolism Decrease circulating lipids	Nausea Diarrhea
Obeticholic acid	FXR agonist	Improve insulin sensitivity Anti-inflammatory Anti-fibrotic	Pruritus Skin rash Abdominal pain

PPAR, peroxisome proliferator activated receptor; SGLT-2, sodium-glucose cotransporter 2; GLP-1, glucagon-like peptide-1; FXR, farnesoid X receptor.
